# Genome-Wide Identification of Microsatellites and Transposable Elements in the Dromedary Camel Genome Using Whole-Genome Sequencing Data

**DOI:** 10.3389/fgene.2019.00692

**Published:** 2019-07-26

**Authors:** Reza Khalkhali-Evrigh, Nemat Hedayat-Evrigh, Seyed Hasan Hafezian, Ayoub Farhadi, Mohammad Reza Bakhtiarizadeh

**Affiliations:** ^1^Department of Animal Breeding and Genetics, Sari Agricultural Sciences and Natural Resources University, Sari, Iran; ^2^Department of Animal Science, University of Mohaghegh Ardabili, Ardabil, Iran; ^3^Department of Animal and Poultry Science, College of Aburaihan, University of Tehran, Tehran, Iran

**Keywords:** *Camelus dromedarius*, *de novo* assembly, repetitive sequence, breeding strategies, next-generation sequencing

## Abstract

Transposable elements (TEs) along with simple sequence repeats (SSRs) are prevalent in eukaryotic genome, especially in mammals. Repetitive sequences form approximately one-third of the camelid genomes, so study on this part of genome can be helpful in providing deeper information from the genome and its evolutionary path. Here, in order to improve our understanding regarding the camel genome architecture, the whole genome of the two dromedaries (Yazdi and Trodi camels) was sequenced. Totally, 92- and 84.3-Gb sequence data were obtained and assembled to 137,772 and 149,997 contigs with a N50 length of 54,626 and 54,031 bp in Yazdi and Trodi camels, respectively. Results showed that 30.58% of Yazdi camel genome and 30.50% of Trodi camel genome were covered by TEs. Contrary to the observed results in the genomes of cattle, sheep, horse, and pig, no endogenous retrovirus-K (ERVK) elements were found in the camel genome. Distribution pattern of DNA transposons in the genomes of dromedary, Bactrian, and cattle was similar in contrast with LINE, SINE, and long terminal repeat (LTR) families. Elements like RTE-BovB belonging to LINEs family in cattle and sheep genomes are dramatically higher than genome of dromedary. However, LINE1 (L1) and LINE2 (L2) elements cover higher percentage of LINE family in dromedary genome compared to genome of cattle. Also, 540,133 and 539,409 microsatellites were identified from the assembled contigs of Yazdi and Trodi dromedary camels, respectively. In both samples, di-(393,196) and tri-(65,313) nucleotide repeats contributed to about 42.5% of the microsatellites. The findings of the present study revealed that non-repetitive content of mammalian genomes is approximately similar. Results showed that 9.1 Mb (0.47% of whole assembled genome) of Iranian dromedary’s genome length is made up of SSRs. Annotation of repetitive content of Iranian dromedary camel genome revealed that 9,068 and 11,544 genes contain different types of TEs and SSRs, respectively. SSR markers identified in the present study can be used as a valuable resource for genetic diversity investigations and marker-assisted selection (MAS) in camel-breeding programs.

## Introduction

Approximately, 42 to 46 million years ago (Mya), ancestors of extant camels appeared in the North America (Honey at al., 1998). The divergence between Camelini (Old World camels) and Lamini (New World camels) occurred in the Early Miocene (Rybczynski et al., 2013). Migration of Old World and New World camel’s ancestor into Eurasia (*via* Bering Isthmus) and South America (*via* Isthmus of Panama), respectively (Heintzman et al., 2015), placed them in two different evolutionary paths. Estimated time for splitting of Old World camels into dromedary and Bactrian camels is approximately 4.4 Mya (Wu et al., 2014). Throughout the past, 4.9 to 7.2 million years (Camelini migration time to Eurasia), Old World camels have become adapted to the deserts of Asia and Africa (Wu et al., 2014) and are used as pack animal for nomads and low-income rural populations (Khalkhali-Evrigh et al., 2018).

Iran is mostly covered by arid or semi-arid regions. Some areas face with increasing population pressure, shortage of water resources, and risk of desertification (Heshmati and Squires, 2013). The evolution has donated some skills to camels enabling them to survive and reproduce in the mentioned condition. Climatic changes and traditional religious and cultural values of Iranians created a high potential for camel breeding in Iran. Currently, dromedary camels are considered as an important supplier of protein for people living in the desert areas in Iran.

It is well known that eukaryotic genome contains a large fraction of repetitive DNA, mainly tandem repeats (satellites, minisatellites, and microsatellites) and interspersed elements [or transposable elements (TEs) (Biscotti et al., 2015). These elements are important components of genomes and are responsible for genome size differences seen across eukaryotes (Sotero-Caio et al., 2017). The sequence, frequency, organization, structure, and location of the repeated units are mainly specific to each species (Pezer et al., 2012).

Since Barbara McClintock discovered TEs (mobile genetic elements) in mid-1940s, many studies have been carried out to understand their genomic function. Generally, TEs are scattered throughout the mammalian genome; however, they are in higher abundance in the heterochromatin (Pardue et al., 1996) and are classified into two classes. Class I (retrotransposon) includes long terminal repeat (LTR) retrotransposon and non-LTR retrotransposon that use an RNA as intermediate to jump (Lerat, 2010), while class II (DNA transposon) uses DNA as intermediate and can be divided into three subclasses including elements that use cut-and-paste, rolling-circle replication (Helitron element), and self-replicating mechanisms (Maverick/Polinton element) for their transposition (Feschotte and Pritham, 2007). Studies revealed that TEs play influential role in regulation of some mammalian gene expression (Medstrand et al., 2005) as well as a source of genetic innovation (Brandt et al., 2005), and also, they contribute in genomes restructuring to enhance the host’s ability to respond to stress (Kidwell and Lisch, 2001).

Microsatellites or simple sequence repeats (SSRs) are known as one of the most variable types of DNA sequences in the genome of many species (Ellegren, 2004). SSRs are randomly distributed across the genome of most eukaryotes; also, they are codominant and highly polymorphic (Abdul-Muneer, 2014). Therefore, microsatellites are informative and widely used for the analysis of genetic diversity within and between populations as well as for evolutionary investigation among the livestock species (Hampton et al., 2004).

Next-generation sequencing technology along with recent advances in assembly strategies made it possible to walk along the genomes and achieve better understanding of them. First whole-genome sequencing of dromedary camel and alpaca (Wu et al., 2014) and Bactrian camels (Jirimutu et al., 2012) has opened a new window for researchers regarding the genome analysis of camelid. Contrary to domesticated species such as cattle, sheep, horse, chicken, and pig, researchers have paid less attention to camels, especially on Iranian camel breeds. Improvement of camel breeding status and designing appropriate breeding schemes require extensive knowledge about camels. Researchers in the light of analysis of camel genome from different aspects can achieve a deep understanding of this species. In this study, we sequenced and assembled (*de novo*) genomes of two Iranian dromedary camels. The distribution and frequency of different TEs and microsatellites were further characterized throughout their genomes. Also, the findings of this study were compared with findings related to other mammalian genomes.

## Materials and Methods

### DNA Extraction and Sequencing

In this study, genomes of two Iranian dromedary camels were sequenced belonging to Yazd station in Yazd Province (YaD) and Trod station in Semnan Province (TrD). Blood samples were taken from Jugular vein and were stored in −20°C till use. DNA extraction was performed using RBC Mini Kit for mammalian blood (Real Biotech Corporation, RBC, South Korea). The extracted DNA was quantified using a NanoDrop, and the identified 260/280 ratio was equal to 1.90 and 1.80 for YaD and TrD, respectively; then quality of the DNA samples was assessed using gel electrophoresis in 1% agarose gel. A library was generated with an average insert size of ∼360 bp, and two lanes of 100 bp paired-end sequencing were carried out using Illumina HiSeq 2000 system (Illumina, San Diego, CA) for each camel.

### Quality Filtering and *de Novo* Assembly

Firstly, FastQC software (https://www.bioinformatics.babraham.ac.uk/projects/fastqc/) was used for quality control of raw sequencing reads. Quality check showed no adapter contamination in raw sequencing reads. Quality filtering of short reads was performed using maximum information (MAXINFO) approach of the Trimmomatic v0.36 (Bolger et al., 2014) with a target length of 40 and strictness value of 0.5. Also, reads with a length less than 40 bp were discarded in the final step of quality filtration. Second, quality control was carried out after quality filtering, and results obtained from FastQC in this step indicated that quality of reads has increased, especially in 3’ end of reads (**Supplementary Figure 1**).

We used CLC Genomics Workbench v9.0 (CLC Bio, Aarhus, Denmark) to perform the *de novo* assembly of trimmed reads using the following parameters: three for mismatch cost, three for insertion and deletion cost, 0.5 for length fraction, and 0.8 for similarity fraction. In order to test the completeness of assembled genomes of our samples and Targui breed (accession number: GCA_001640815.1), BUSCO strategy was applied using *Mammalia* (containing 4,104 genes) and *Vertebrata* (containing 2,586 genes) datasets. In fact, BUSCO uses sets of Benchmarking Universal Single-Copy Orthologs from OrthoDB (www.orthodb.org) to report completeness degree of assemblies (Simao et al., 2015). Also, we used MUMmer3 (nucmer script) for comparison of our genomes with existing dromedary reference (accession number: GCA_000803125.1); results are presented in the **Supplementary File 1** (YaD with reference) and file2 (TrD with reference).

### TEs Identification

Homology-based approach was used to identify TEs in assembled genomes of Iranian dromedaries. RepeatMasker v4.0.7 program (http://www.repeatmasker.org) was used to search for known TEs using the combination of Repbase v20170127 and Dfam databases. Repbase is a comprehensive database of repetitive sequences from human and other eukaryotic organisms. We used RMBlast v2.6.0 as a sequence search engine for RepeatMasker with Smith-Waterman cutoff 255 (based on Fitak et al., 2016). It is worth mentioning that RMBlast is a RepeatMasker compatible version of the standard NCBI BLASTn program, which is used to search for repeats. Also, *de novo* identification of TEs in genomes of Iranian dromedaries was performed using RepeatModeler v1.0.11 program (http://www.repeatmasker.org/RepeatModeler). In fact, RepeatModeler assists in automating the runs of RECON (Bao and Eddy, 2002) and RepeatScout (Price et al., 2005) as two *de novo* repeat finding programs, to analyze our genomes.

### SSRs Identification

Assembled genomes were searched for identifying the microsatellites using MIcroSAtellite identification tool (MISA, http://pgrc.ipk-gatersleben.de/misa/) with motif size ranging from mono-nucleotide to octo-nucleotide. The minimum repeat numbers were defined as 12 for mono-, 6 for di-, 5 for tri- and tetra, 4 for penta- and hexa-, and 3 for hepta- and octo-nucleotide repeat SSRs. Microsatellite motifs that interrupted by 100 nucleotides were considered as compound microsatellites. Also, several mammalian assembled genomes were downloaded and searched for microsatellite loci, including Arabian dromedary camel, Bactrian camel, alpaca, horse, cattle, sheep, and human (downloaded from NCBI Reference Sequence Database, RefSeq). Our goal was to produce a dataset to compare with the results obtained for Iranian dromedaries. In order to extract comparable results, discovery of SSRs was done for all genomes with same parameters. Accession numbers of downloaded assembled genomes are presented in **Supplementary Table 1**.

### Annotation of Repeats

We employed MAKER v2.31.10 pipeline (Cantarel et al., 2008; Holt and Yandell, 2011) to annotation of Iranian dromedary genome (YaD). It is a powerful tool for annotation of newly sequenced genome and also updating existing annotations. Protein and mRNA sequences for dromedary and Bactrian camels were obtained from NCBI as input for MAKER to homology-based gene identification. MAKER aligns these sequences to newly assembled genome for discovery of putative genes. CD-HIT (CD-HIT-2d; Li and Godzik, 2006) was used to prepare a database from dromedary and Bactrian protein sequences, as input for MAKER. In fact, CD-HIT-2d compares two protein datasets and one of the similar sequences in each cluster at a certain threshold (95% identity in our work) is reported as output. Finally, BEDtools v2.25.0 (Quinlan, 2014) was performed to find genes containing the TEs and SSRs. Based on the 80–80–80 rule (Wicker et al., 2007), genes containing TEs shorter than 80 bp were filtered out. It should be mentioned that DAVID v.6.8 (Huang et al., 2008) was used for functional enrichment analysis of genes containing some class of TEs (all subfamilies of MIRs) and SSRs. The calculated p-values were corrected using the Benjamini correction for multiple testing, and enriched terms were considered statistically significant at p-adjusted < 0.1.

## Results

### *De Novo* Assembly and Completeness Assessment of Iranian Dromedary Genome

Sequencing of the samples using Illumina HiSeq 2000 platform in paired end yielded a total of 920,366,954 (92 Gb) and 843,455,144 (84.3 Gb) raw reads for YaD and TrD, respectively. Filtering to a threshold of Q20 sequence quality produced 899,714,102 and 826,229,484 clean reads, which were *de novo* assembled using CLC Genomics Workbench assembler. The clean reads were applied to assemble the genome for each camel, separately. The assembly process yielded 137,772 and 149,997 contigs with total consensus genome size of 1.94 Gb in YaD and TrD. The contig N50 length was equal to 54.6 and 54 kb for YaD and TrD, respectively, indicating good quality assembly for further downstream analysis (**Table 1**). The averaged GC contents of the assembled genome were 41.52% and 41.58% in YaD and TrD, respectively, which was slightly higher than the reported GC content for African dromedary (41.3%; Fitak et al., 2016), Arabian dromedary (41.2%), alpaca (41.4%; Wu et al., 2014), and wild Bactrian camel (41.28%; Jirimutu et al., 2012). The results of completeness test on assembled genomes using BUSCO revealed that 93.7% of the 4,104 genes in the *Mammalia* dataset were present in YaD (74.1% complete genes, 19.6% fragmented genes) and TrD (73.9% complete genes, 19.8% fragmented genes) genomes. Also, *Vertebrata* dataset was used to investigate the completeness of assemblies, and it was found that 94.9% and 94.8% of vertebrate genes were present in the YaD and TrD assemblies, respectively.

**Table 1:** Summary of the YaD and TrD genome assembly.

Contigs	YaD	TrD
N25 (bp)	97,128	95,611
N50 (bp)	54,626	54,031
N75 (bp)	26,694	26,620
Longest contig	466,683	604,268
Average contig length (bp)	14,101	12,980
Counts of contigs	137,772	149,997
Total bases (Gb)	1.94	1.94

### Identification of Repeats in Iranian Dromedary Genomes

The amount of repetitions of the assembled genomes was assessed in order to annotate and determine how their components are organized, including TE and SSR diversity, distribution, and dynamics. To do this, a *de novo* and homology-based approach was applied to identify microsatellites and TEs using the assembled genomes, respectively. Based on RepeatMasker outputs, totally, 30.58% of Yazdi and 30.5% of Trodi camel genomes were composed of TEs. However, due to high similarity of repetitive sequences in two Iranian dromedary camels as well as similar assembled genome sizes of both, average values of different repetitive elements were used for discussion (**Table 2**). In fact, results of homology-based method on repeats identification showed that the total length of TEs content was equal to 594.1 Mb (30.54% of assembled genome) in YaD and TrD camels. Also, results of *de novo* based identification of TEs for YaD and TrD, as additional information for future studies, are presented in **Supplementary Table 2**.

**Table 2:** Summary of identified transposable elements for Iranian dromedaries, African dromedary, and Bactrian camel.

TEs	Iranian dromedary			African dromedary		Bactrian camel	
	Numbers	Length (bp)	%	Numbers	%	Numbers	%
**SINEs**	**458,654**	**68,422,832**	**3.51**	**473,387**	**3.43**	**560,273**	**3.92**
Alu/B1	0	0	0.00	7	0.00	0	0.00
MIRs	452,075	67,605,477	3.48	463,927	3.38	460,330	3.38
**LINEs**	**838,990**	**354,299,228**	**18.22**	**1,009,426**	**19.28**	**883,074**	**19.32**
LINE1	483,215	260,189,224	13.38	642,633	14.57	552,674	14.82
LINE2	301,928	81,743,253	4.20	312,130	4.10	280,625	3.92
L3/CR1	39,626	8,761,454	0.45	40,821	0.44	38,504	0.44
RTE	13,046	3,412,114	0.18	–	–	10,913	0.14
**LTRs**	**286,303**	**103,155,112**	**5.31**	**324,636**	**5.43**	**321,595**	**5.81**
ERVL	82,052	35,068,299	1.80	80,984	1.72	76,625	1.63
ERVL-MaLRs	140,115	47,792,407	2.45	138,020	2.35	133,362	2.31
ERV-classI	39,386	13,849,285	0.71	81,938	1.07	77,025	1.63
ERV-classII	0	0	0.00	571	0.00	23,095	0.10
**DNA elements**	**331,140**	**68,223,263**	**3.50**	**341,448**	**3.44**	**282,696**	**3.00**
hAT-Charlie	188,154	36,658,424	1.88	186,819	1.79	175,700	1.68
TcMar-Tigger	53,998	14,327,829	0.74	66,902	0.81	44,496	0.68
**Total**	**2,581,776**	**625,581,334**	**30.54**	**2,905,840**	**31.58**	**2,628,996**	**32.05**
Reference	Present study			Fitak et al. (2016)		Jirimutu et al. (2012)	

Results of TE annotation revealed that 635 types of them were located in 9,068 genes (**Supplementary Table 3A**). LINEs with 50,653 copies had the most distribution in the genic regions followed by SINEs, LTRs, and DNA transposons with 28,701, 16,839, and 11,442 copies, respectively. Some TEs such as Eulors and UCONs (173 elements) were grouped as unclassified elements. About 28,444 of identified genic SINEs belonged to all subfamilies of MIRs (MIR, MIRb, MIRc, MIR3, and MIR1_Amn) that have influenced 6,920 genes.

### Profiles of SSRs

**Figure 1:**
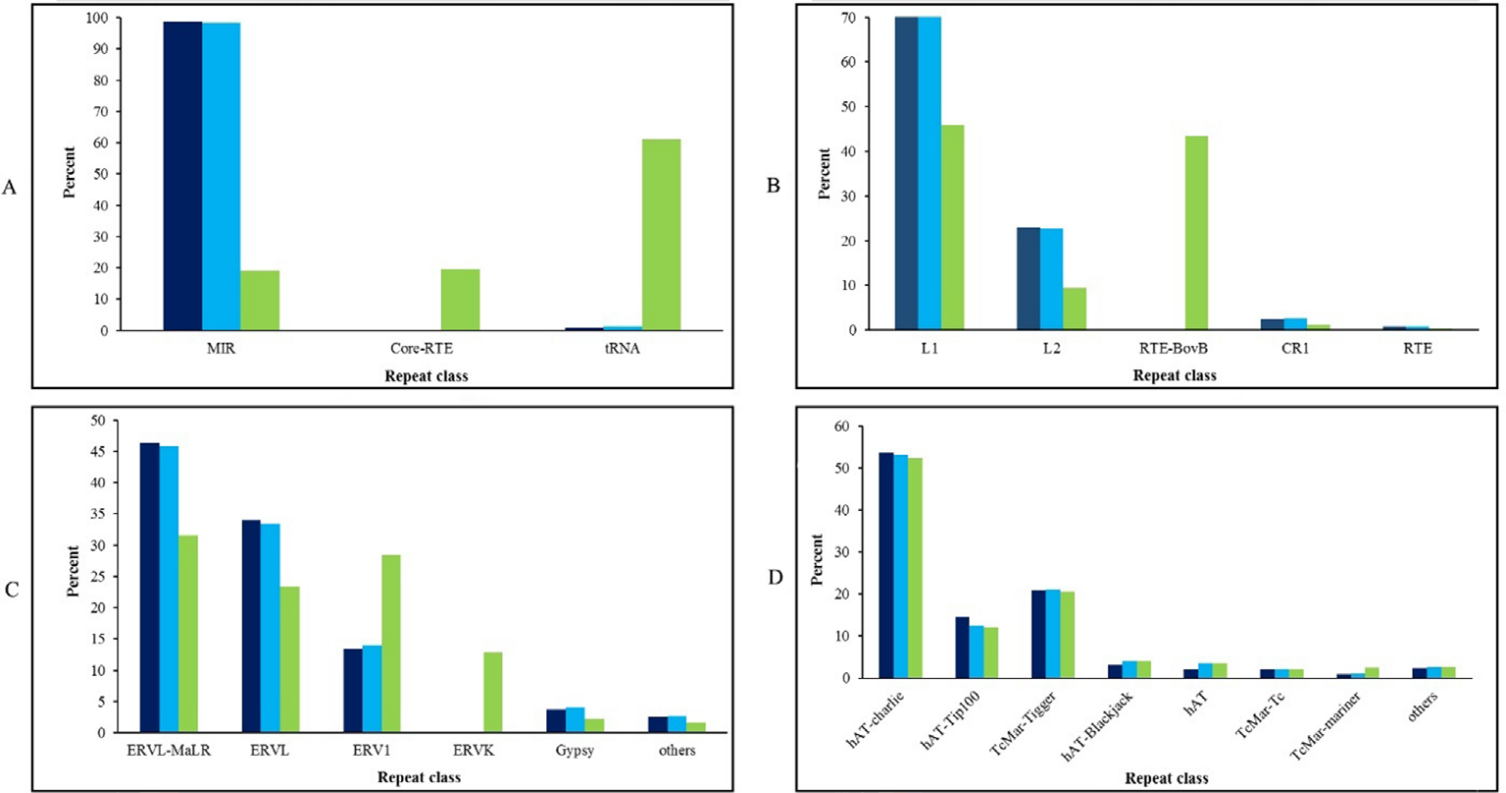
Frequency of different SSR motifs across YaD, TrD, and other seven mammalian genomes.

SSRs are extremely useful molecular markers for study on genomic diversity among individuals as well as among populations and different species (Saeed et al., 2016). Therefore, genome-wide identification of these makers can be considered as a valuable genomic resource for population characterization. We investigated the SSR distribution on Iranian dromedaries as well as on the seven mammalian genomes. Frequencies of different types of microsatellite in dromedary camels and other mammals are presented in **Figure 1** and **Table 3**. The highest and lowest percentages of SSRs in whole-genome content belong to human (0.79% of assembled genome) and horse (0.32% of assembled genome), respectively.

**Table 3:** Distribution of different classes of SSRs in YaD, TrD, and other seven mammalian genomes.

Species	Mono	Di	Tri	Tetra	Penta	Hexa	Hepta	octo	Total (bp)
YaD	3,670,743	3,429,832	638,283	781,068	329,445	111,366	97,839	59,368	9,117,944
TrD	3,669,900	3,417,918	636,564	778,964	325,930	110,706	97,790	58,208	9,095,980
Arabian dromedary	4,121,293	3,859,778	685,755	1,390,196	401,970	129,516	97,986	89,456	10,775,950
Bactrian camel	4,082,129	3,791,158	615,105	1,147,212	366,725	125,718	99,785	92,632	10,320,464
Alpaca	2,065,967	4,102,030	627,525	1,100,084	348,420	123,546	152,278	72,032	8,591,882
Cattle	5,045,498	4,858,676	2,046,501	254,296	1,537,845	54,444	94,374	26,576	13,918,210
Sheep	4,657,293	4,889,182	1,770,183	392,980	1,589,625	62,922	78,764	57,312	13,498,261
Horse	3,344,142	3,025,388	577,437	532,720	180,225	47,934	64,001	37,848	7,809,695
Human	12,287,665	6,754,988	1,537,530	3,001,280	1,439,420	291,282	304,525	149,072	25,765,762

The results of scanning the assembled genomes revealed the presence of 86,121 and 69,061 contigs possessing microsatellite motifs in YaD and TrD, respectively. Totally, 540,133 and 539,409 microsatellites loci were found, corresponding to 9.1 Mb of repeat bases which represents 0.47% of the total bases of the genomes in both Iranian dromedary genomes. Among these, 50,805 and 50,896 sequences belonged to compound types in YaD and TrD, respectively. The frequency of microsatellite repeats was found to range from one motif per 2,435.1 bp in human to one motif per 5,147.9 bp in horse genome. For YaD and TrD, the density of SSRs was equal to one motif per 3,596.8 and 3,609.4 bp, respectively, indicating that camel genome has a medium SSR density compared to the investigated mammalian genomes.

Results of SSR annotation showed that there are 42,636 SSRs in the 11,544 genes (**Supplementary Table 3B**). Tri- and hexa-nucleotide SSRs include ∼10% of all genic SSRs. As expected, the most SSRs in genic regions belonged to mono- (21,512 motifs) followed by di-nucleotide (12,831 motifs) SSRs. Results of gene ontology (GO) analysis for 1,000 genes with the largest number of all types of SSRs revealed that these genes are present in places such as membrane and cell cortex. Also, we found that terms such as ATP binding, ATPase activity, ligase activity, etc. were significantly enriched in molecular function category (**Supplementary Table 4A**).

## Discussion

As a prerequisite to genomic scale studies as well as development of breeding programs in Iranian dromedaries, we used the first whole-genome resequencing data of individual camels from two distinct geographical regions (Khalkhali-Evrigh et al., 2018) for mining of repetitive content. Contig N50 lengths obtained in present study were found to be longer than African dromedary (40.2 kb; Fitak et al., 2016) and Targui breed dromedary (31.5 kb; GenBank accession: GCA_001640815.1) but shorter than Arabian dromedary (69.1 kb; Wu et al., 2014) and wild Bactrian camel (90.3 kb; Jirimutu et al., 2012). The results of completeness test related to previously published assembled genome in contig level (BioProject: PRJNA310822; Targui breed) revealed that, despite the various libraries in mentioned project, better completeness was achieved in this study compared to Targui breed (**Supplementary Table 5**). The shorter assembled genomes (1.94 Gb) in present study, compared to 2.01 Gb in Arabian dromedary (Wu et al., 2014) and 2.08 Gb in Targui dromedary, may be attributed to the lack of libraries with long insert size in the present study. However, results of BUSCO implied that our genome assembly can be comparable to previously reported assemblies.

Identification of the amount of repetitions regions in genomes can be used in refining genome assembling and annotation. Furthermore, proper annotation of repeats provides information on the evolutionary mechanisms involved in species differentiation and how they diversified over the evolutionary process (Mehrotra and Goyal, 2014). Results of RepeatMasker for TEs investigation in Iranian dromedary genome were found to be close to African breed dromedary (31.58%; Fitak et al., 2016), Bactrian camel (30.37%), and alpaca (32.14%; Wu et al., 2014), but less than horse (47.3%; Adelson et al., 2010) and cattle genomes (46.5%; Adelson et al., 2009). This number of repetitive regions in these genomes can be attributed to C-value paradox, which is the observed lack of correlation between increases in DNA content and an organism’s complexity. In other words, there is a correlation between genome size in eukaryotes and repetitive regions (not gene content) (Eddy, 2012). Therefore, higher repetitive content of horse, cattle, and human genomes may be due to their bigger genome size.

Previous findings showed that lengths of non-repetitive regions of mammalian genomes are similar, as cattle (genome size of 2.67 Gb), sheep (genome size of 2.61 Gb), horse (genome size of 2.47 Gb), dromedary camel (genome size of 2.05 Gb), and Bactrian camel (genome size of 1.99 Gb) have 1.37, 1.41, 1.25, 1.32, and 1.36 Gb unique genomic content, respectively. Here, non-repetitive content of assembled genomes was 1.32 Gb for both Iranian dromedary genomes that are in agreement with other mammalian genomes. Seemingly, calculated non-repetitive regions in mammalian genomes are conserved section of their genomes; however, proof of this claim requires further studies.

In the present study, TEs were found to contribute in 30.54% of assembled genome of Iranian dromedary camels. It is reported that LINEs and SINEs are very old TEs in mammalian genomes (about thousands of millions of years ago) (Medstrand et al., 2005). L1 is mostly located in AT-rich regions and is dominant LINE in mammalians, while LINE2 (L2) is uniformly distributed throughout genome (Gu et al., 2000). Also, abundance of LINEs within genes is less than their abundance in upstream and downstream regions of genes. On the other hand, SINEs are overrepresented within genes in comparison with LINEs (Medstrand et al., 2005). In the present study, we found that LINE elements were the most prevalent interspersed repeats and contributed 354.3 Mb (18.22%) of the total assembled genomes, which was slightly lower than African dromedary (19.28%) and Bactrian camel (19.32%). Additionally, in LINE class, LINE1 (L1) and RTE elements with 483,215 (13.38%) and 13,046 (0.18%) copies were the most and least frequent elements, respectively. It is well known that LINE RTE (BovB) elements are considered as the gift of squamates to ruminants. Presumably, horizontal transfer of BovB elements has been done by ticks (especially *Amblyomma* and *Bothriocroton* species) as common vectors between squamates, ruminants, monoterms, and African mammals (Walsh et al., 2013). BovB elements comprised 10.70% and 11.70% of cattle (Adelson et al., 2009) and sheep genome, respectively, while this value for dromedary camel, Bactrian camel, horse, and pig were 0.035%, 0.051%, 0.079%, and 0.034%, respectively. Further studies are needed to understand why distribution pattern of BovB elements is different throughout domesticated mammalian genomes.

Content of SINEs was less than LINEs and comprised only 3.51% of the total genome length. It is well known that Alu elements are mostly enriched in GC-rich or gene-rich regions, and they are considered as abundant and conserved repeat family in primate genomes (Gu et al., 2000). In this study, no Alu elements were found in the Iranian dromedary camel genomes. This finding was in agreement with Bactrian camels; however, Fitak et al. (2016) reported seven Alu sequences in African dromedary camel. Due to the lack of mentioned seven Alu annotations in African dromedary camel genome, we were unable to perform specific similarity searches. Therefore, we cannot determine if the Iranian dromedary camel’s genome actually lacks Alus or if this is a false-negative result due to the sequence divergence of Alu elements of camels and primates.

Mammalian-wide interspersed repeats (MIRs) are another important member of SINE family. Positive association has been found between existence of one TE in genic regions and tissue-specific gene expression for MIRs (Jjingo et al., 2011). Jjingo et al. (2014) studied on human genome and revealed that MIRs are rich source of transcription factor binding sites compared to random genomic regions. Therefore, enrichment of MIRs within enhancers influences gene expression level as well as tissue-specific gene expression. We found that 67.6 Mbp (3.48%) of Iranian dromedary camel genome was covered by MIRs, which was slightly higher than Bactrian camel and African camel (3.38%). Study on MIR elements in RNA-seq level can help us to better understand the roles of MIRs in camel genome. Among all the TE elements identified in Iranian dromedary camel genome, 286,303 copies were classified as LTR elements including ERVL, ERVL-MaLRs, ERV-classI, and ERV-classII (**Table 2**).

**Figure 2:**
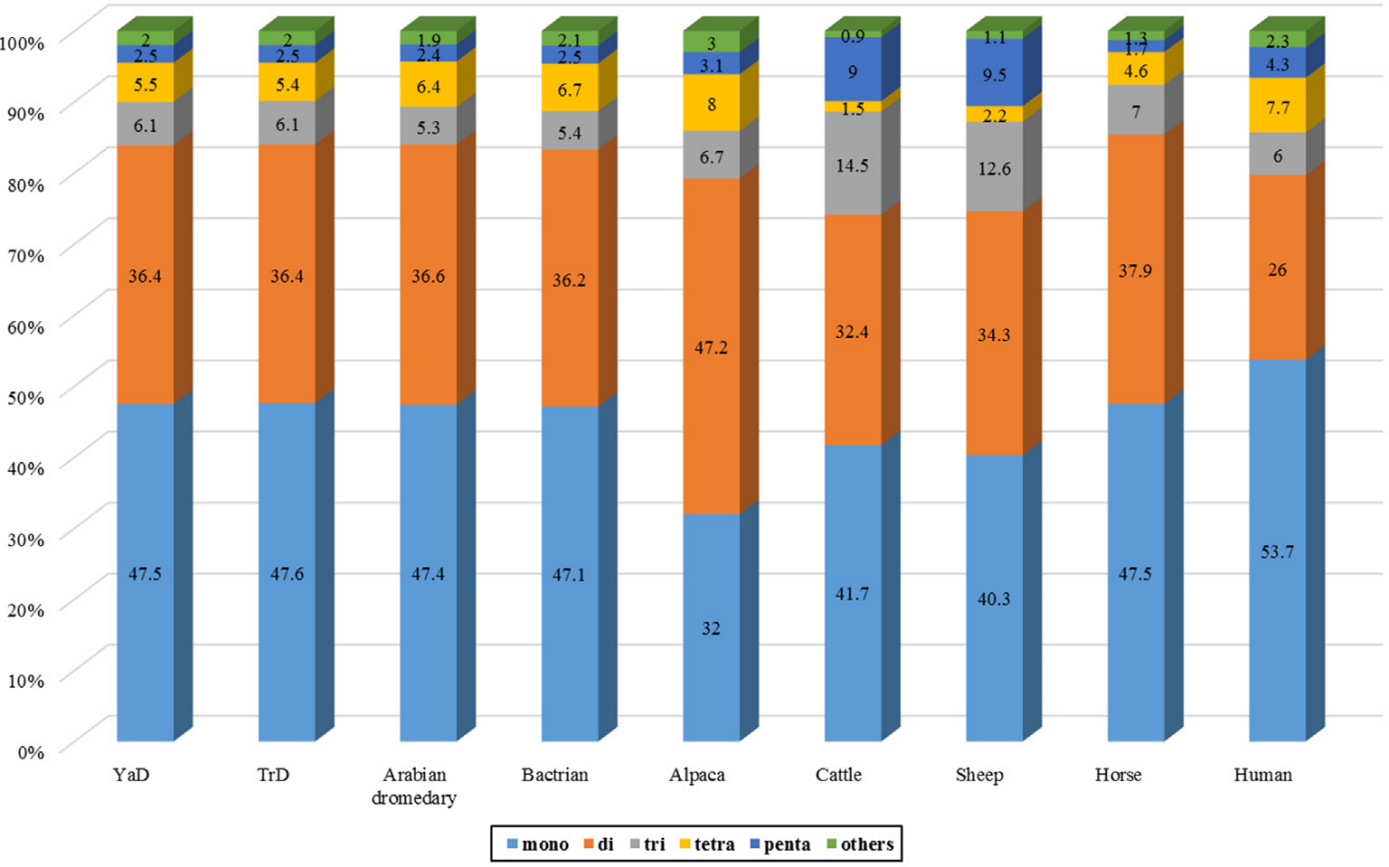
The content of SINE **(A)**, LINE **(B)**, LTR **(C)**, and DNA transposon elements **(D)** in the genomes of Iranian dromedaries (dark blue), Bactrian camel (blue), and cattle (green).

The content of different interspersed repetitive sequence families including SINEs, LINEs, LTRs, and DNA transposons in dromedary, Bactrian, and cattle genomes is shown in **Figure 2**. A distinct pattern was observed in the proportion of each subfamily elements of SINEs, LINEs, and LTRs for cattle genome compared to camelid genomes. Mammalian LTR transposon (MaLR) was the most frequent member of LTRs in all three genomes. MaLR elements [with 1.5–10-kb length (Gu et al., 2000)] were inserted in mammalian genome about 70 million years ago (Bènit et al., 1999). The previous findings about TEs in horse genome showed that most of MaLR elements in genic regions were located in coding region (96 elements) in comparison with 3’-UTR (6 elements) and 5’-UTR (0 element) (Ahn et al., 2013). Comparison of DNA elements in dromedary, Bactrian, and cattle genome showed that hAT-charlie element is the most abundant DNA elements among all three genomes.

It has been shown that 0.05% and 0.07% of cattle and horse genomes are composed of ERVK (belonging to LTRs class) elements, respectively (Adelson et al., 2009; Adelson et al., 2010). However, ERVK element was absent in dromedary’s genome that is in accordance with African dromedary (Fitak et al., 2016) and Bactrian camel (Jirimutu et al., 2012). ERVKs are one of the youngest members of endogenous retroviruses (ERVs) family (Katoh and Kurata, 2013), and the conservation of the specific protein binding site in some ERVKs probably reflects the regulatory role of them for the nearby located genes in the human genome (Akopov et al., 1998). Unlike the LINE, SINE, and LTR families, distribution of DNA transposon elements was similar among dromedary, Bactrian, and cattle.

In this study, we found that 3.5% of dromedary genomes belong to DNA transposons, in which hAT-charlie and TcMar-Tigger elements were the most abundant members of this family. Percentages of DNA elements in the genomes of human, mouse, cattle (Adelson et al., 2009), horse (Adelson et al., 2010), and Bactrian camel (Jirimutu et al., 2012) are 3, 0.89, 1.96, 3.1, and 3, respectively. Scientists believed that the last activity of DNA transposons in mammalian genomes has occurred at least 40 million years ago. However, Ray et al. (2008) provided evidences for recent activity of hAT and Helitron elements in bat (*Myotis lucifugus*) lineage. These results pave the way for further investigation of active elements in mammalian genomes as well as their effects.

**Figure 3:**
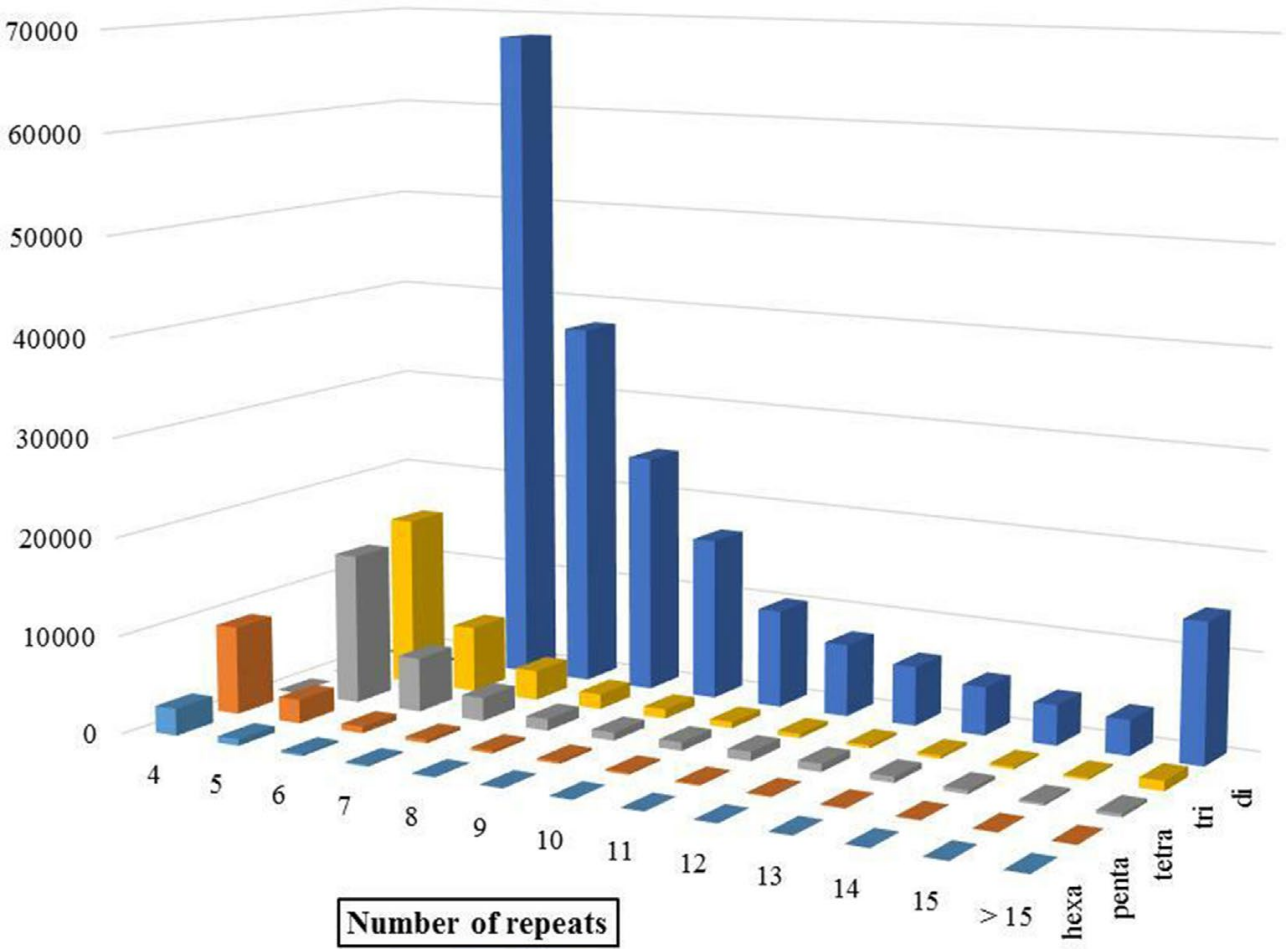
Distribution of different SSR motifs in Iranian dromedary genome.

Discovery and mining of genomic repeats such as SSRs are considered as a successful approach in genetic analysis, linkage mapping, and marker-assisted breeding (Pandey et al., 2017). The results of assessment of microsatellite distribution in whole genome of Iranian dromedaries revealed that the number of microsatellites decreased with the increase in size. Also, for each class of repeats, the number of motifs decreased as the number of repeats increased (**Figure 3**). For example, di-nucleotide motif with six repeats contains 34.4% (67,724) of total di-nucleotides, while this value for 15 repeats was equal to 1.8% (3,610). Number of the identified microsatellite motifs ranged from 469,380 (alpaca) to 1,336,255 (human). SSR contents in YaD and TrD were lower than those of Arabian dromedary (10.7 Mb) and Bactrian camel (10.3 Mb). One possible explanation for the discrepancy among SSR contents is different length and assembly levels of the genomes; as in this study, the genomes were assembled at contig levels, but Arabian and Bactrian camel genomes were made at the scaffold level. Unexpectedly, pattern of microsatellites in alpaca, as a camelid, was different from dromedary and Bactrian camels. Mono-nucleotide (47.1% to 47.6%) was the most frequent motif in Old World camels, whereas di-nucleotide motifs (47.2%) were dominant in alpaca. The results revealed that lowest and highest mono-nucleotide motifs belonged to alpaca (32%) and human (53.7%), respectively. The highest content of tetra-nucleotide motifs was assigned to alpaca followed by human, Old World camels, horse, sheep, and cattle.

(T)n motifs were found to be the most abundant repeat in YaD, TrD, Bactrian camel, cattle, sheep, horse, and human, whereas (A)n was the most abundant motif for Arabian dromedary and alpaca. Among di-nucleotide SSRs, AC/GT type was enriched in the genomes followed by AT/TA and AG/CT types in all under study mammalians. In case of tri-nucleotide SSRs, around 26–28% of them belonged to AAT/ATT in camelid, whereas shares of AAT/ATT motifs in horse and human genome were 25.76% and 39.91%, respectively. The highest percentage of tri-nucleotide motifs in sheep and cattle genome belonged to AGC/CTG motif with a share of about 68.3% and 75.51% of all tri-nucleotide motifs, respectively. The AAAC/GTTT motif was the most frequent tetra-nucleotide motif in dromedaries and Bactrian camel, while the AAAT/ATTT motif had the highest number of tetra-nucleotide in others. In the case of hexa-nucleotide, AAAAAC/GTTTTT motif was the most replicated motif in all genomes except cattle genome (**Supplementary File 3**). Assessment of most frequent motifs in different classes of SSRs revealed that there is a high similarity between mammalians in this regard. This similarity along with non-random distribution of SSRs throughout genome and functional roles of them (Vieira et al., 2016) may reflect the importance of these repeated sequences in evolution process in mammalians.

Results of genome-wide identification of microsatellites in Iranian dromedaries revealed that AT-rich motifs were dominant in all classes of repeats. In this context, A/T motifs in mono-nucleotide, AC/GT in di-nucleotide, AAC/GTT in tri-nucleotide, AAAC/GTTT in tetra-nucleotide, AAAAC/GTTTT in penta-nucleotide, AAAAAC/GTTTTT in hexa-nucleotide, AAAAAAC/GTTTTTT in hepta-nucleotide, and AAAAAAAC/GTTTTTTT in octo-nucleotide were found as the most abundant motif type.

Furthermore, 10 abundant microsatellite motifs with highest frequencies for each genome were studied. Totally, it was found that more than 74% of all microsatellites in each genome belonged to top 10 frequent motifs (**Table 4**). Also, a very similar pattern of these microsatellites was observed among dromedaries, Bactrian camel, alpaca, horse, and even human. In mentioned species, all of 10 motifs were composed of mono- and di-nucleotide.

**Table 4:** 10 SSRs with most frequency in YaD, TrD, and other seven mammalian genomes.

Rank	YaD	TrD	Arabian dromedary	Bactrian camel	Alpaca	Cattle	Sheep	Horse	Human
1	T	T	A	T	A	T	T	T	T
2	A	A	T	A	T	A	A	A	A
3	AC	AC	AC	AC	AC	TG	TG	TG	AC
4	TG	TG	TG	TG	TG	AC	AC	AC	TG
5	AT	AT	AT	AT	AT	AT	AT	TA	AT
6	TA	TA	TA	TA	TA	AGC	TA	CA	TA
7	GT	GT	GT	GT	GT	TA	CA	AT	GT
8	CA	CA	CA	CA	CA	CA	GT	GT	CA
9	TC	TC	TC	TC	TC	GT	AGC	TC	TC
10	AG	AG	AG	AG	AG	TGC	ACTGA	AG	AG
From all (%)	78.81	78.83	79.06	79.02	74.96	76.15	74.27	78.54	77.29

Then, unique SSR loci were considered and human was found as the most diverse species with 5,544 SSR types. For alpaca, the number of SSR motif types was equal to 5,296 followed by TrD (5,122), YaD (5,112), Bactrian camel (4,995), Arabian dromedary (4,853), horse (3,869), sheep (3,597), and cattle (3,223). Also, 1,629 SSR motifs were identified in common between camelid genomes. Moreover, 847 motif types were identified, which were shared among all evaluated genomes in this study (**Supplementary Table 6**). Species-specific motifs in camelid genomes were 782 (17 in tetra-, 101 in penta-, 195 in hexa-, 296 in hepta-, and 173 in octo-nucleotide). On the other hand, 209 motifs (5 tetra-, 65 penta-, 25 hexa-, 77 hepta-, and 37 octo-nucleotide) were found in common among human, cattle, sheep, and horse, whereas they were not observed in camelid genomes (**Supplementary Table 7**).

Generally, the findings of the present study showed that the content and distribution of the identified repetitive regions were similar (not the same) among Iranian dromedaries and the other camel breeds with sequenced genome. The differences in the repetitive content of these breeds can be attributed to different factors, such as different evolutionary origin or discrepancy in the assembly stage of these genome projects. Of note, repetitive sequences are much harder to assemble in a *de novo* manner and tend to form smaller contigs, resulting in different content of repetitive distribution in closely related breeds.

Because of the potential regulatory role of MIRs in mammalian genomes (Wang et al., 2015), GO analysis was applied on genes containing this class of TEs. For this, 1,000 genes were selected with the most number of any subfamilies of MIRs for classification based on biological process, cellular component, and molecular function. For these genes, we found no significantly enriched GO term in biological process and cellular component but two for molecular function including ATP binding and calcium ion binding (**Supplementary Table 4B**). Optimum energy metabolism is vital for camels due to the low food and harsh living environment of them; therefore, the presence of these elements in the genes related to energy metabolism in camel genome because of regulatory roles and possible link between MIRs and enhancers (Jjingo et al., 2014) is considered an interesting result. Absolutely, more and deeper studies are needed to prove the relevance of MIRs with important genes (like genes associated with energy metabolism) in the camel genome.

Here, for the first time, DNA sequencing technology was used for *de novo* genome assembly of Iranian dromedaries. Although the amount of applied sequencing data was not adequate for whole-genome assembly, it could help us to obtain an overview regarding the repetitive elements in the genome. Furthermore, in order to generate a comprehensive annotation of the Iranian dromedary camels’ repeatome, we used a computational approach. The results revealed that, on average, 594.1 and 9.1 Mb of Iranian dromedary’s genome length are made up of TEs and SSRs, respectively.

The finding of this study will be applied as a valuable resource for further studies on camel breeding, especially on Iranian dromedary’s breeds. The large number of camel’s SSR markers developed in this study established a valuable resource for investigation of genetic diversity, marker-assisted selection (MAS) and may improve the development of breeding programs in Iranian dromedary camels in the future. This study was like shedding light on a part of the camel genome. Absolutely, conduction of more studies would provide more information and awareness about genomic features of camels. Increasing genome-wide information about camels could improve the designed strategies used for its maintenance and breeding.

## Ethics Statement

All animal care and experiments were approved by the animal science committee of the University of Mohaghegh Ardabili, Iran. Also, all experiments were performed in accordance with a routine guideline, which is acceptable by this committee. It is worth to mention that, for reducing stress of animals, positive rewards such as petting were implemented in the conditioning to regular handling prior to restraint for blood collection.

## Author Contributions

RK-E and MRB developed the idea and analyzed the data. NH-E collected the samples and obtained funding and required equipment for the project. SHH supervised the project, AF advised the project, and RK-E, NH-E, and MRB interpreted the results and wrote the manuscript.

## Conflict of Interest Statement

The authors declare that the research was conducted in the absence of any commercial or financial relationships that could be construed as a potential conflict of interest.

The handling editor and reviewer POtW declared their involvement as co-editors in the Research Topic, and confirm the absence of any other collaboration.

## Supplementary Material

The Supplementary Material for this article can be found online at: https://www.frontiersin.org/articles/10.3389/fgene.2019.00692/full#supplementary-material. Also, some extra supplementary files including outputs of RepeatMasker, RepeatModeler, MISA and annotation of repeats, are archived in the Dryad Digital Repository under doi: 10.5061/dryad.10h185k
