# Supplementary material for: Genome-Wide Identification of Microsatellites and Transposable Elements in the Dromedary Camel Genome Using Whole-Genome Sequencing Data
Source: Front Genet. 2019 Jul 26;10:692. doi: 10.3389/fgene.2019.00692 (PMC6675863; doi:10.3389/fgene.2019.00692)
Supplement: Supplementary file 1 [file Table_1.docx]

| **Supplementary Table 1** Accession numbers of studied mammalian genomes in present work | | | | |
| --- | --- | --- | --- | --- |
| Source | Accession number | Genome size (Gb) | Scientific name | Common name |
| Refseq | GCF_000001405.37 | 3.2 | *Homo sapiens* | Human |
| Refseq | GCF_000767585.1 | 2 | *Camelus dromedarius* | Dromedary camel |
| Refseq | GCF_000767855.1 | 2 | *Camelus bactrianus* | Bactrian camel |
| Refseq | GCF_000164845.2 | 2.2 | *Vicugna pacos* | Alpaca |
| Refseq | GCF_000003055.6 | 2.7 | *Bos taurus* | Cow |
| Refseq | GCF_000298735.2 | 2.6 | *Ovis aries* | Sheep |
| Refseq | GCF_000002305.2 | 2.5 | *Equus caballus* | Horse |
