# Supplementary material for: Genome-Wide Identification of Microsatellites and Transposable Elements in the Dromedary Camel Genome Using Whole-Genome Sequencing Data
Source: Front Genet. 2019 Jul 26;10:692. doi: 10.3389/fgene.2019.00692 (PMC6675863; doi:10.3389/fgene.2019.00692)
Supplement: Supplementary file 2 [file Table_2.docx]

| **Supplementary Table 2** Summary of de-novo based identified transposable elements for Iranian dromedary camels | | | | | | | |
| --- | --- | --- | --- | --- | --- | --- | --- |
| TEs | YaD | | |  | TrD | | |
|  | Numbers | Length (bp) | % |  | Numbers | Length (bp) | % |
| **SINEs** | **315246** | **43529244** | **2.24** |  | **311913** | **41199423** | **2.12** |
| Alu/B1 | 0 | 0 | 0.00 |  | 0 | 0 | 0.00 |
| MIRs | 228561 | 32335956 | 1.66 |  | 216792 | 29869429 | 1.53 |
| **LINEs** | **725765** | **256740753** | **13.22** |  | **749758** | **258343151** | **13.27** |
| LINE1 | 597864 | 230935236 | 11.89 |  | 613269 | 234762983 | 12.06 |
| LINE2 | 124223 | 24920895 | 1.28 |  | 128875 | 22417224 | 1.15 |
| L3/CR1 | 2290 | 239730 | 0.01 |  | 6679 | 610768 | 0.03 |
| **LTRs** | **280752** | **82212597** | **4.23** |  | **301357** | **84491697** | **4.34** |
| ERVL | 72426 | 24897990 | 1.28 |  | 80919 | 24399789 | 1.25 |
| ERVL-MaLRs | 116404 | 31758944 | 1.63 |  | 130726 | 34003313 | 1.75 |
| ERV-classI | 91284 | 25437213 | 1.31 |  | 88524 | 25749956 | 1.32 |
| ERV-classII | 638 | 118450 | 0.01 |  | 643 | 134225 | 0.01 |
| **DNA elements** | **225886** | **38392419** | **1.98** |  | **238512** | **40309816** | **2.07** |
| hAT-Charlie | 126042 | 21250264 | 1.09 |  | 126411 | 22386808 | 1.15 |
| TcMar-Tigger | 48753 | 9530961 | 0.49 |  | 45104 | 9212596 | 0.47 |
| **Unclassified** | **29851** | **10171185** | **0.52** |  | **30608** | **10010738** | **0.51** |
| **Total** | **1577500** | **431046198** | **22.19** |  | **1632148** | **434354825** | **22.31** |
