# Supplementary material for: Genome-Wide Identification of Microsatellites and Transposable Elements in the Dromedary Camel Genome Using Whole-Genome Sequencing Data
Source: Front Genet. 2019 Jul 26;10:692. doi: 10.3389/fgene.2019.00692 (PMC6675863; doi:10.3389/fgene.2019.00692)
Supplement: Supplementary file 4 [file Table_4.docx]

| **Supplementary Table 4A:** Results of gene ontology (GO) analysis on 1000 genes containing the most SSRs in YaD camel | | | | | |
| --- | --- | --- | --- | --- | --- |
| **Category** | **GO term** | **Description** | **Count** | **p-value** | **Benjamini** |
| CC | GO:0070062 | extracellular exosome | 163 | 4.12E-08 | 2.05E-05 |
| CC | GO:0016020 | membrane | 72 | 9.64E-05 | 0.0158823 |
| CC | GO:0005938 | cell cortex | 14 | 7.00E-05 | 0.0172776 |
| CC | GO:0005925 | focal adhesion | 31 | 6.68E-04 | 0.0798729 |
| MF | GO:0005524 | ATP binding | 119 | 7.42E-12 | 4.75E-09 |
| MF | GO:0016887 | ATPase activity | 18 | 2.10E-05 | 0.0067029 |
| MF | GO:0016874 | ligase activity | 16 | 8.45E-05 | 0.0178739 |
| MF | GO:0005509 | calcium ion binding | 51 | 1.55E-04 | 0.0244716 |
| MF | GO:0003777 | microtubule motor activity | 11 | 5.06E-04 | 0.0627282 |
| MF | GO:0004842 | ubiquitin-protein transferase activity | 22 | 6.82E-04 | 0.0702144 |
| MF | GO:0044822 | poly(A) RNA binding | 65 | 9.66E-04 | 0.0846088 |

| **Supplementary Table 4B:** Results of gene ontology (GO) analysis on 1000 genes containing the most MIR elements in YaD camel | | | | | |
| --- | --- | --- | --- | --- | --- |
| **Category** | **GO term** | **Description** | **Count** | **p-value** | **Benjamini** |
| MF | GO:0005524 | ATP binding | 103 | 9.14E-07 | 6.67E-04 |
| MF | GO:0005509 | calcium ion binding | 56 | 7.04E-06 | 0.002565 |
