# Supplementary material for: Genome-Wide Identification of Microsatellites and Transposable Elements in the Dromedary Camel Genome Using Whole-Genome Sequencing Data
Source: Front Genet. 2019 Jul 26;10:692. doi: 10.3389/fgene.2019.00692 (PMC6675863; doi:10.3389/fgene.2019.00692)
Supplement: Supplementary file 5 [file Table_5.docx]

| **Supplementary Table 5** Results of completeness test for de novo assembled genomes of YaD, TrD and previously published assembled targui breed genome using mammalia and vertebrata datasets | | | | | | | | |
| --- | --- | --- | --- | --- | --- | --- | --- | --- |
| Targui breed | |  | TrD | |  | YaD | |  |
| Vertebrata | Mammalia |  | Vertebrata | Mammalia |  | Vertebrata | Mammalia |  |
| 1739 | 2658 |  | 1951 | 3012 |  | 1958 | 3023 | Complete and single-copy |
| 29 | 31 |  | 21 | 20 |  | 20 | 17 | Complete and duplicated |
| 631 | 982 |  | 482 | 812 |  | 476 | 804 | Fragmented |
| 187 | 433 |  | 132 | 260 |  | 132 | 260 | Missing |
| 2586 | 4104 |  | 2586 | 4104 |  | 2586 | 4104 | Total |
