# Supplementary material for: Genome-Wide Identification of Microsatellites and Transposable Elements in the Dromedary Camel Genome Using Whole-Genome Sequencing Data
Source: Front Genet. 2019 Jul 26;10:692. doi: 10.3389/fgene.2019.00692 (PMC6675863; doi:10.3389/fgene.2019.00692)
Supplement: Supplementary file 11 [file Image_1.pdf]

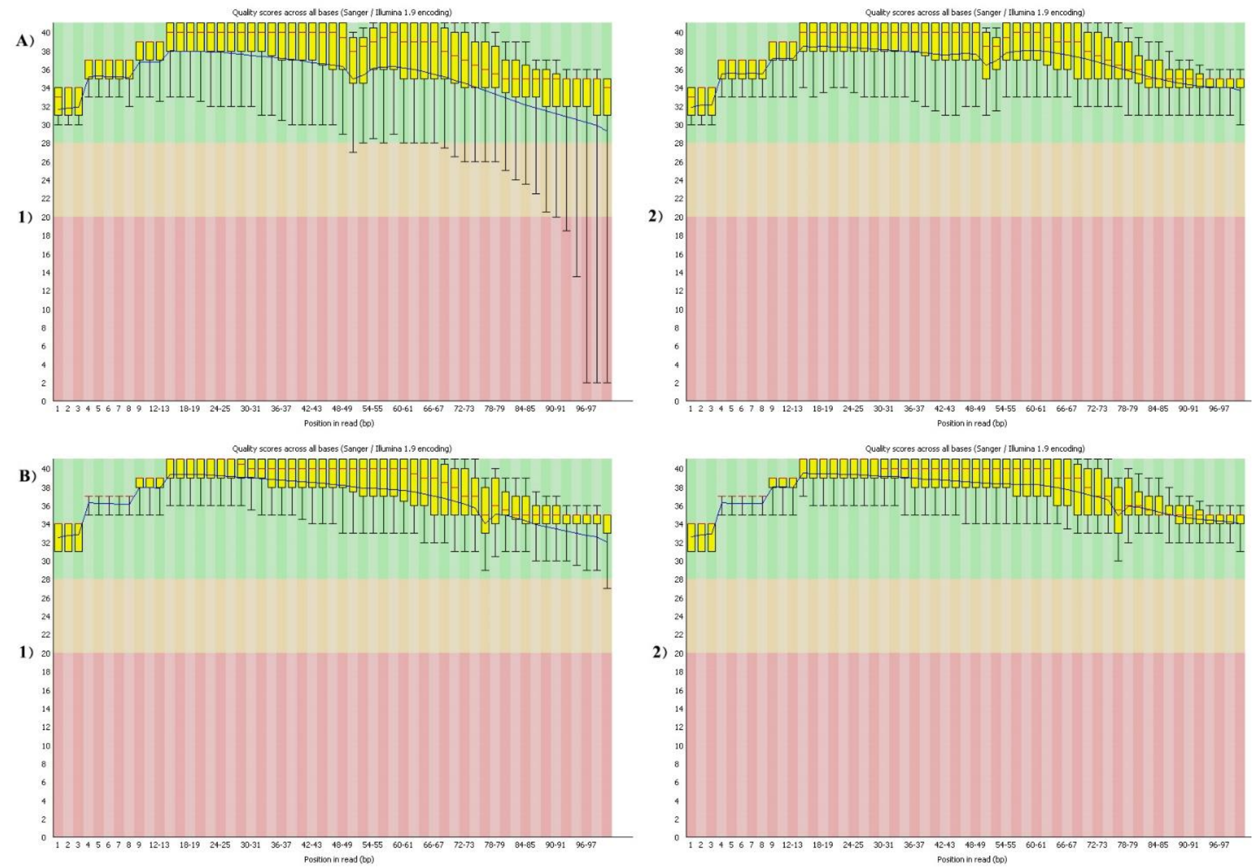

Supplementary Figure 1 Quality reports of reads for YaD (A) and TrD (B), before (1) and after (2) quality filtration.
